# Use of a Low Concentration, High Volume Erector Spinae Plane Block for Rescue Analgesia After Melanoma Resection and Axillary Sentinel Lymph Node Biopsy

**DOI:** 10.7759/cureus.12930

**Published:** 2021-01-27

**Authors:** Aaron R Muncey, Raymond Evans, Allan R Escher, Daniel A Nahrwold

**Affiliations:** 1 Anesthesiology, Moffitt Cancer Center, Tampa, USA

**Keywords:** erector spinae plane block, postoperative pain control, regional anesthesia, rescue analgesia, ultrasound, axillary surgery

## Abstract

The erector spinae plane block (ESPB), a recent innovation in regional anesthesia, has been used for analgesia of the thorax and chest. The case presented describes the use of an ESPB postoperatively for rescue analgesia on an elderly, opioid-naïve patient, who had severe postoperative pain after outpatient surgery at an axillary sentinel lymph node biopsy site refractory to escalating IV opioid doses. The rescue ESPB was successful in reducing the patient’s pain to 0/10, allowing the patient to be discharged home and preventing a costly hospital admission.

## Introduction

The ESPB has been increasingly used as a regional analgesic modality since it was introduced in 2016 as an alternative approach for patients with thoracic neuropathic pain from rib fractures or metastases [1]. Since then, application of the ESPB has expanded to include planned postoperative and rescue analgesia for thoracic [2], breast [3-7], abdominal [8], and orthopedic surgery [9]. It continues to be studied as an analgesic modality for treatment of rib fractures and flail chest as in its original presentation [6, 10]. 

Previous case reports and randomized controlled trials have detailed use of the ESPB for breast surgery including axillary sentinel lymph node biopsy, axillary dissection, and axillary clearance [3-5, 7]. Only one case report exists describing the use of the ESPB in isolated axillary surgery [11]. And no case reports describe the use of the ESPB for rescue analgesia after isolated axillary surgery. This case is unique in that the ESPB was used for rescue analgesia on an elderly opioid-naïve patient, who experienced severe postoperative pain refractory to significant IV opioids after a planned outpatient left upper extremity melanoma resection with deep axillary sentinel lymph node biopsy. 

## Case presentation

A 73-year-old, 91.8 kg white woman with a past medical history of poorly controlled hypertension, obesity (BMI 34.9), osteoarthritis, and hyperlipidemia presented to the Moffitt Cancer Center outpatient surgery center for radical resection of a left upper arm melanoma (1.9 mm) with complex layered wound closure and left axilla deep sentinel lymph node biopsy. The patient’s home medications included meloxicam 15 mg po bid and ibuprofen 800 mg po prn for left knee osteoarthritis and 0.25 mg alprazolam po bid prn anxiety, but no opioids. She was a lifelong nonsmoker with no history of recreational drug use who consumed alcohol socially two to four drinks per month.

In the preoperative bay, the patient received 1,000 mg po acetaminophen and 200 mg po celecoxib. After receiving 2 mg of midazolam prior to the operating room, the patient was preoxygenated and induced with 150 mg of IV propofol by slow push, preceded my 50 mcg of IV fentanyl and 50 mg of IV 2% lidocaine. A size 4 laryngeal mask airway (LMA) was placed easily in one attempt and confirmed with end-tidal CO2 and bilateral breath sounds. Surgery took 46 min from 8:48 am to 9:34 am during which the patient received an additional 50 mcg of IV fentanyl and postoperative nausea prophylaxis with 8 mg of IV dexamethasone and 4 mg of IV ondansetron. The patient emerged from anesthesia uneventfully with LMA removal at 9:37am and transfer to post-anesthesia care unit (PACU) at 9:42 am.

In PACU, at 9:55 am the patient noted no pain at the left upper extremity incision site but significant pain at the left sentinel axillary node biopsy site with a severity of 8/10 on the numeric pain scale (0-10). She was given 0.2 mg IV hydromorphone in repeated doses starting at 9:57 am until 10:20 am for a total of 1 mg (five doses) of IV hydromorphone. Her pain scores measured every 5 min remained 8/10 until 10:20 am. At 10:27 am, her pain score decreased to 7/10, and she received an additional 0.4 mg (two doses) of IV hydromorphone, which further reduced her pain score to 6/10 by 10:45 am. She received an additional 0.4 mg (two doses) of IV hydromorphone between 10:45 am and 11:45 am, at which time her measured pain score remained 6/10. The patient received in total 100 mcg of IV fentanyl and 1.8 mg of IV hydromorphone. The surgeon was notified of the patient’s persistent severe pain by the PACU nurse and initiated plans to transport the patient by ambulance to Moffitt Main Campus and admit for poorly controlled postoperative pain. 

**Figure 1:**
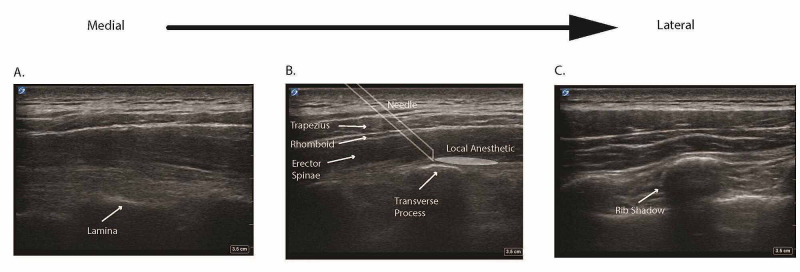
Overview of ultrasound anatomy for the erector spinae block scanning medial to lateral. A.  When starting medially, the first key structure identified by ultrasound is the lamina, which appears as a deep hyperechoic line.  B.  Moving laterally, the transverse process, more superficial than lamina, is identified as a hyperechoic line with a square shadow.  Above T5, the trapezius, rhomboid, and erector spinae muscles appear as three layers with hyperechoic lines above the transverse process.  Local anesthetic is injected between the erector spinae muscle and transverse process.  C.  Appearance of circular shadows indicates the ultrasound is above the ribs, lateral to the transverse processes.

The PACU nurse then notified the anesthesiologist who decided to perform a left-sided ultrasound (US) guided ESPB for persistent postoperative pain. The patient was prepped with chlorhexidine and draped. The T4 transverse process was identified on US by counting from cephalad to caudad and scanning medial to lateral (Figure 1).

The needle was advanced caudally in-plane until touching the T4 transverse process as visualized on US, then pulled back slightly to allow injection of 30 mL of 0.25% ropivacaine with intermittent aspiration every 5 mL. The US confirmed hydrodissection beneath the erector spinae muscle. The patient tolerated the procedure well and 15 min later reported 0/10 pain. Plans for overnight admission and transport via ambulance were cancelled, and the patient was discharged home.

She did not require readmission, and at a follow up clinic visit on postoperative day (POD) 11, reported the block wore off in the evening of POD 1, at which time her pain was 5/10 and tolerable with acetaminophen-hydrocodone 325 mg-5 mg prescribed by the surgeon. The patient noted persistent throbbing left axillary pain extending to the left pectoralis muscle rated 5/10, worse at night and improved by holding her arm close to her body. Even 11 days postoperatively, the pain was still significant and interfered with her daily functioning. 

## Discussion

The ESPB holds promise for many applications including thoracic, breast, abdominal, and orthopedic surgery, trauma, and chronic neuropathic pain [1-3, 5, 7-10]. In this case, it was successful as rescue analgesia for refractory pain in the axilla after deep sentinel lymph node biopsy. The case was unique in that an elderly opioid-naïve woman, with no significant recreational drug or alcohol use, developed such severe pain from sentinel node biopsy despite 100 mcg of IV fentanyl and 1.8 mg IV hydromorphone, which were administered by standard incremental dosing orders based on the procedure and patient. It is conceivable the patient might have suffered an inadvertent neuropathic injury during the surgical biopsy, given that pain persisted at a relatively high level (5/10) even 11 days postoperatively. 

Conflicting reports exist on the extent of analgesia provided by the ESPB and a recent cadaver study demonstrated variable spread of injectate upon surgical dissection after US guided ESPB. Eleven different cadavers demonstrated variable dermatomal spread of the same volume of injectate. Several cadavers had involvement of dorsal rami, ventral rami, or the paravertebral space and four cadavers showed spread to the contralateral side [12]. In our case a low concentration, high volume strategy was employed by using 30 mL of 0.25% ropivacaine given this known heterogeneity. The result was complete analgesia which prevented a costly hospital admission by ambulance transfer. A recent study where ESPB was performed on 12 healthy volunteers found that in addition to variable dermatomal spread as in the cadaver study, the ESPB only provided cutaneous sensory loss to the posterior thorax, and in no volunteers did the sensory block extend ventrally beyond the posterior axillary line. The authors suggested that this is likely due to local anesthetic block of the dorsal rami of the spinal nerves [13]. In that study 20 mL of 0.5% ropivacaine was used compared with 30 mL of 0.25% ropivacaine in this case. This suggests that a high volume, low concentration approach may be more suitable when analgesia anterior to the posterior axillary line is desired. 

However, there are likely patient-specific anatomic factors involved. In a recent case report, an elderly 73 kg woman with cardiac disease received 20 mL of 0.5% ropivacaine with epinephrine 1:200,000 and dexamethasone 8 mg for a T5 ESPB, which produced complete surgical anesthesia for a radical mastectomy with axillary dissection [5]. The patient weight was lower than in our case, and additives (epinephrine and dexamethasone) were employed. Another case series using the ESPB for breast and axillary surgery found strong analgesic effects with three different strategies all using 0.5% ropivacaine with 2.5 μg/mL epinephrine: 20 mL using a T3 ESPB, 15 mL for a T2 ESPB plus 15 mL for a T4 ESPB on the same side, and 15 mL for a T4 ESPB [7]. Another case series of three patients undergoing total mastectomy with sentinel/axillary node dissection used 30 mL of 0.375% ropivacaine with epinephrine 1:200,000, a higher volume strategy similar to ours but at a higher concentration and with epinephrine. They placed catheters and provided a bolus of the same volume and concentration every 12 h and found that patients required only one to two bolus demand doses of a fentanyl patient-controlled analgesia (PCA) in three days [3].

The only known case of an ESPB for isolated axillary surgery used a similar strategy of low concentration, high volume with 30 mL of 0.25% bupivacaine hydrochloride with 3 mg of dexamethasone and produced complete analgesia in a patient with hidradenitis suppurativa [11]. In this case the ESPB was performed at T2 compared with our T4.

A recent meta-analysis of randomized controlled trials of ESPB in breast surgery found a significant reduction in opioid consumption and pain scores 24 h postoperatively compared with general anesthesia, but inferior to the pectoralis nerve block (PECS) block [4]. Included studies had variable local anesthetics, concentrations, injection volumes, and dermatomal injection levels. This variability and inclusion of surgeries with no axillary involvement make it difficult to ascertain the significance of ESPB for axillary pain. 

## Conclusions

Our case study demonstrated significant analgesic benefit using a low concentration high volume strategy for the ESPB as rescue analgesia for axillary surgery and suggests this is a reasonable approach in patients with refractory axillary pain postoperatively. As a single-patient case report this study is limited by nature and future studies are needed to determine the efficacy of this approach in the broader patient population as well as optimal local anesthetic, concentration, volume, additives, and dermatomal injection level for ESPBs involving surgery of the axilla.
